# Nutrient recycling and utilization of *Torreya grandis* ‘Merrillii’ along an age gradient

**DOI:** 10.3389/fpls.2025.1566140

**Published:** 2025-04-17

**Authors:** Aifei Fan, Songheng Jin, Yangzhou Tan, Weiwei Huan, Wenjing Chen, Xiaoyu Wang, Yini Han

**Affiliations:** ^1^ Jiyang College, Zhejiang A&F University, Zhuji, China; ^2^ School of Forestry and Biotechnology, Zhejiang A&F University, Hangzhou, China; ^3^ School of Art and Design, Nanjing Vocational University of Industry Technology, Nanjing, China

**Keywords:** stand age, *Torreya grandis*, stoichiometry, nutrient resorption, nutrient recycling

## Abstract

**Introduction:**

The intrinsic relationships among plants, litter, and soil nutrient characteristics, along with the responses of ecological stoichiometry to nutrient utilization, are critical for understanding the mechanisms of nutrient cycling. However, limited research in this area has constrained our comprehension of nutrient dynamics within ecosystems.

**Methods:**

To investigate the stoichiometric characteristics and nutrient resorption traits of Torreya grandis plantations across various stand ages, as well as their adaptive strategies and nutrient utilization mechanisms under local growth conditions, we conducted a study in the *T. grandis* Forest Park. This study examined five stand age groups: young (20 years), near-mature (50 years), mature (80 years), over-mature (100 years), and thousand (1,000 years). We measured the nutrient contents of soil, fresh leaves, and litterfall, and analyzed their stoichiometric relationships and nutrient resorption characteristics.

**Results:**

1.The growth of *T. grandis* plantations was primarily limited by nitrogen (N) during the early stages, transitioning to phosphorus (P) limitation with increasing stand age, particularly in the over-mature stage. High C:N and C:P ratios in leaves indicated low N and P use efficiency. 2.Leaf nutrient concentrations remained relatively stable across different stand ages, whereas nutrient concentrations in litterfall gradually declined, indicating an increase in nutrient cycling efficiency. Meanwhile, soil nutrient accumulation showed a gradual increase with stand development. *T. grandis* exhibited distinct nutrient resorption strategies at different stand ages: phosphorus resorption efficiency (PRE) was higher in young stands, whereas nitrogen resorption efficiency (NRE) significantly increased in mature and over-mature stands. Furthermore, this nutrient allocation mechanism influenced the nutritional content of *T. grandis* seeds, highlighting the significant impact of stand age on seed quality. 3.The nutrient characteristics of *T. grandis* plantations are influenced by both stand age and soil nutrient availability.Management practices should prioritize the supplementation of soil nutrients, particularly P, and the enhancement of nutrient cycling efficiency.

**Discussion:**

This study offers a scientific foundation for the sustainable management and production of *T. grandis* plantations in the region, highlighting the importance of targeted soil nutrient management to improve ecosystem productivity and sustainability.

## 1 Introduction

Ecological stoichiometry investigates the mass balance of multiple nutrient elements across scales, from individual organs to entire ecosystems (Zhu et al., 2020). This approach has been extensively applied to analyze ecosystem functions and nutrient limitations, providing novel perspectives on the complex dynamics of nutrient cycling within ecosystems (Bai et al., 2020). The fundamental nutrient elements, including Carbon(C), nitrogen(N), and phosphorus (P), are crucial for plant metabolism and growth processes (Elser et al., 2010; Huang et al., 2019; Wang et al., 2019). The stoichiometric ratios of these elements in various ecosystem components are often used to describe key ecological processes and explore the interdependence of elemental cycles (Hattenschwiler and Jorgensen, 2010; Wang et al., 2020). These ratios serve as effective tools for analyzing and interpreting the relationships and variations between plants and their environments within ecosystems (Zeng et al., 2016). They are frequently used to identify nutrient limitation types, evaluate the coupling among multiple nutrients, and assess plant adaptation to diverse environmental conditions (Tian et al., 2019). Soil plays a crucial role in nutrient supply, with the soil C:N:P ratio serving as an indicator of soil fertility and influencing both nutrient absorption and nutrient limitations in plants (Fan et al., 2015; Liu et al., 2016; Bai et al., 2019). The C, N, and P status of leaves is closely linked to essential aspects of plant growth, reproduction, and ecosystem functioning. These traits can serve as indicators of how plants utilize nutrients and respond to environmental changes (Qin et al., 2019). Leaf stoichiometric characteristics are considered to reflect plant genetic traits and their adaptations to specific environmental conditions (Bai et al., 2020). Litterfall, as a nutrient reservoir (Xiao et al., 2015), has a C:N:P ratio that significantly influences the quality and rate of nutrient recycling within ecosystems (Xiao et al., 2015; Zhang XM et al., 2017).

The C:N:P stoichiometry of soil and plants can indicate nutrient limitations in ecosystems. Specifically, a high C:N ratio or low N:P ratio is typically associated with N limitation, whereas high C:P or N:P ratio often indicates P limitation. Furthermore, the C:N:P stoichiometry of soil and plants is closely related to nutrient-use efficiency in plants (Elser et al., 2010). When plant growth is constrained by limited N or P availability, plants adopt adaptive strategies, such as adjusting the C:N:P ratios at the organ level, to address nutrient constraints (Ajmera et al., 2022; Huang et al., 2023). Some plants adopt nutrient conservation strategies in low-P environments, maintaining stable leaf P concentrations and P allocation to adapt to nutrient-poor habitats (Fan et al., 2024). Furthermore, under N-limited conditions, plants may preferentially mineralize inorganic N from the soil; under P-limited conditions, they are more likely to enhance root exudation or modify root architecture to acquire more P (Wang et al., 2022). Thus, examining the C:N:P stoichiometric characteristics of the leaf-litter-soil continuum is crucial for understanding nutrient limitations in plants and nutrient cycling processes in terrestrial ecosystems (Dong et al., 2020).

Stand age can significantly alter soil physicochemical properties, microclimatic conditions, litterfall input, and root exudates. These changes, in turn, influence microbial communities and soil enzyme activities, making stand age a critical factor in determining soil nutrient distribution (Hedo et al., 2016; Yesilonis et al., 2016). Due to substantial differences in photosynthetic capacity and nutrient demands at different growth stages, nutrient stoichiometry and nutrient resorption in plantations often exhibit temporal variations (Yan et al., 2018). Understanding how plant nutrient stoichiometry and resorption efficiency change over time has been a major focus of research, yet the findings remain inconsistent (Zhou et al., 2016). For instance, studies have reported that the C:N ratio of plant tissues in global secondary forests increases significantly with stand development (Yang and Luo, 2011). However, research on the N availability in developing *Nothofagus solandri* var. cliffortioides (Hook. f.) Poole forests found no significant changes over time (Clinton et al., 2002). In coastal Chinese plantations of *Metasequoia glyptostroboides*, the C:P and N:P ratios of young stands were significantly lower than those of middle-aged stands (Zhang et al., 2018). From young to mature stands, NRE and PRE gradually increased (Sun et al., 2016) whereas both NRE and PRE remained stable across different stand ages in Japanese larch (*Larix kaempferi*) forests (Chang et al., 2017). Such discrepancies may stem from the element-specific characteristics of nutrients or species-specific responses of trees (Wang et al., 2014). Changes in the concentrations of nutrients (C, N, and P) reflect the nutrient uptake and utilization strategies of stands of varying ages and their adaptability to environmental conditions (Yang and Luo, 2011). The relationships between plant stoichiometric ratios and stand age have significant implications for plantation management and fertilization strategies. Consequently, exploring the interplay between stand age and plant adaptability to diverse environmental conditions is of considerable research value.

The leaf N:P ratio is widely recognized as a critical indicator for diagnosing N or P limitations in terrestrial ecosystems (Zhang et al., 2018). Previous studies have shown that during the chronological development of artificial forests, such as *Larix kaempferi*, *Metasequoia glyptostroboides*, and *Robinia pseudoacacia*, the leaf N:P ratio significantly increased from below 14 to above 16, indicating a shift from relative N limitation to relative P limitation over time (Yan et al., 2018). These findings suggest that, as stand age progresses in subtropical plantations, tree growth likely transitions from N limitation to P limitation. Similar nutrient limitation trends are also observed across different ecosystems worldwide (Elser et al., 2007). In studies related to nutrient limitations in the Amazon forest, it has been observed that secondary forests growing on abandoned agricultural lands initially exhibit N limitation. However, as forest succession progresses, there is a gradual transition to P limitation. This transition reflects the changes in N and P cycling associated with forest development over time (Davidson et al., 2007). However, research on this transition in subtropical plantations remains limited, as tree growth in these ecosystems is often constrained by low soil P availability. Moreover, the adaptive mechanisms of trees to simultaneous N and P limitations remain unclear (Chang et al., 2017).

*Torreya grandis* ‘Merrillii’, an endemic and rare nut tree species in China, has been cultivated for over 1,000 years (Li et al., 2005). To guide high-yield cultivation and promote the sustainable development of the *T. grandis* industry, extensive research has been conducted on cultivar improvement, seedling propagation, and management practice (Yan et al., 2021).While some studies have investigated soil nutrient conditions and the interaction between *T. grandis* of various ages and soil nutrients (Zhang et al., 2019; Dong et al., 2021), research on nutrient cycling within the “leaf-litter-soil” continuum in *T. grandis* plantation ecosystems is scarce. Nutrient availability has been identified as a critical factor influencing both yield and quality in *T. grandis* plantations. In recent years, the planting area of *T. grandis* has expanded due to its high ecological and economic value. Consequently, a large number of *T. grandis* stands at different growth stages are present in the study region, yet the nutrient utilization strategy, nutrient requirements, and the mechanism of nutrient resorption in leaves and litter of *T. grandis* across different stand ages remain unclear. It is generally believed that seed yield and quality increase with stand age. From the perspective of nutrient utilization and cycling, we hypothesize that the nutrient utilization and transformation capacity of *T. grandis* do not decline with increasing stand age, and we aim to explain this based on the nutrient utilization and absorption mechanisms of *T. grandis* across different stand ages. This study aims to elucidate the stoichiometric characteristics, nutrient limitation patterns, nutrient resorption efficiency, and nutrient cycling processes within the *T. grandis* plantation ecosystem. To achieve this, leaf, litter, soil, and nut samples were collected from *T. grandis* plantations spanning five age groups: 20 years (young), 50 years (near-mature), 80 years (mature), 100 years (over-mature), and 1,000 years (ancient). The nutrient contents and their dynamic changes were systematically analyzed. The objectives of this study are: (1) To investigate the C, N, and P contents and stoichiometric characteristics of leaves, litter, and soil in *T. grandis* plantations; (2) To examine how the C, N, and P contents and stoichiometry within the leaf-litter-soil continuum vary with stand age; (3) To reveal the nutrient absorption and utilization traits across different stand ages in *T. grandis* plantations. The findings of this study aim to provide scientific support for the management and conservation of *T. grandis* plantations in the region.

## 2 Materials and methods

### 2.1 Study area

The study site is located in the National *T. grandis* Forest Park (119°53′01″~120°32′08″ E, 29°21′24″~29°59′05″ N), at the foothills of Kuaiji Mountain in Zhaojia Town, Zhuji City, Zhejiang Province. This area is situated in the inland area of central Zhejiang and falls within the subtropical monsoon climate zone, characterized by typical hilly and mountainous climate features. The average annual temperature is 16.3°C, with an average annual precipitation of approximately 1373.6 mm, and the average number of precipitation days per year is about 158.3. The soil types in the study area are mainly hilly red soil and river valley plain paddy soils, with a sandy loam texture. The study site has been used for the cultivation and management of *T. grandis* artificial forests for many years.

### 2.2 Sampling and measure methods

The study site is located in a plantation area of *T. grandis*, which has been managed using the same management practices for decades. The site has a rich age gradient composition, and this study selected five age gradients young, near-mature, mature, over-mature, and thousand in relatively flat areas with similar altitudes for sampling and analysis. Plant Sampling: Three representative *T. grandis* trees of similar growth in each age group were randomly selected. Fresh and senescent leaves were collected from the middle of the canopy, stored in refrigerated conditions, and transported to the laboratory for measurements of fresh and dry weights. Soil Sampling: In each age group, three soil sampling points were randomly placed along a diagonal line within the planting area. After removing the surface litter, soil samples were collected from the 0–10 cm and 10–20 cm soil layers, stored in refrigerated conditions, and transported to the laboratory. After removing roots and stones, the samples were air-dried and passed through a 2 mm sieve. Dried leaf and air-dried soil samples were ground into fine powders for the determination of C, N, and P concentrations. Fruit Sampling: Ten fully mature and non-cracked fruits (with seeds intact) were randomly collected from the upper middle part of the south side of each tree canopy. After being washed, the outer aril of *T. grandis* seeds was manually removed. The seeds were then subjected to blanching at 90°C for 30 minutes using an oven, followed by drying at 60°C for 24 hours until a constant weight was achieved for further parameter analysis.

### 2.3 Chemical element measurement

The C and N contents in plant samples, as well as the N content in soil, were determined using an elemental analyzer (Vario MACRO cube, Elementar, Germany) based on the combustion method (Dieckow et al., 2007). SOC content was determined using the potassium dichromate external heating method by Heanes (Heanes, 2008). Plant P and soil TP and AP contents were measured using the molybdenum blue colorimetric method (Nelson and Sommers, 1982) All chemical analyses were performed with three sample replicates, and the concentrations of C, N, and P in the samples were expressed as mass concentrations. Measurement of content indicators in *T. grandis* Seeds: The samples were dried to constant weight (the difference between two consecutive measurements did not exceed 0.002 g), shelled, and then the seeds were ground evenly for subsequent analysis. The oil content was determined using the GB/T 14772-2008; protein content was measured using the GB 5009.5-2010 “Determination of Protein in Foods”; fatty acid components were determined using GB 5009.168-2016 “Determination of Fatty Acids in Foods”. The protein content of seeds was determined using the Kjeldahl method in accordance with GB 5009.5-2016 (Yang, 2009).

### 2.4 Data analysis

The soil C, N, and P contents represent the soil organic carbon, total nitrogen, and total phosphorus content, respectively. The C, N, and P stoichiometric ratios are expressed as mass ratios, namely C:N, C:P, and N:P. The abbreviations for green leaves, senescent leaves, and soil are G, L, and S, respectively.

The nutrient contents and stoichiometric ratios of leaves, litter, and soil of *T. grandis* were analyzed using Repeated-Measures Analysis of Variance (ANOVA) using the SPSS Statistics 26 software (SPSS Inc., Chicago, IL, USA). All of the treatment means were compared for any significant differences using Duncan’s Multiple Range Test (DMRT) or Welch’s ANOVA to determine the trends in the variations of C, N, and P, their stoichiometric characteristics, and the nutrient content of seeds across different ages. Pearson correlation analysis was conducted on the C, N, P, and C:N:P ratios in leaves, litter, and soil, as well as nutrient resorption. Redundancy Analysis (RDA) was conducted using CANOCO 5.0 software to investigate the relationships between leaf-litter-soil C, N, P stoichiometric ratios and soil physicochemical properties. Linear regression was applied to analyze nutrient resorption rates of N and P in leaves. Data plotting was performed using Origin 2021 software (Origin Lab Inc., Northampton, MA, USA).

## 3 Result

### 3.1 Nutrients contents and stoichiometric characteristics of leaf

**Figure 1:**
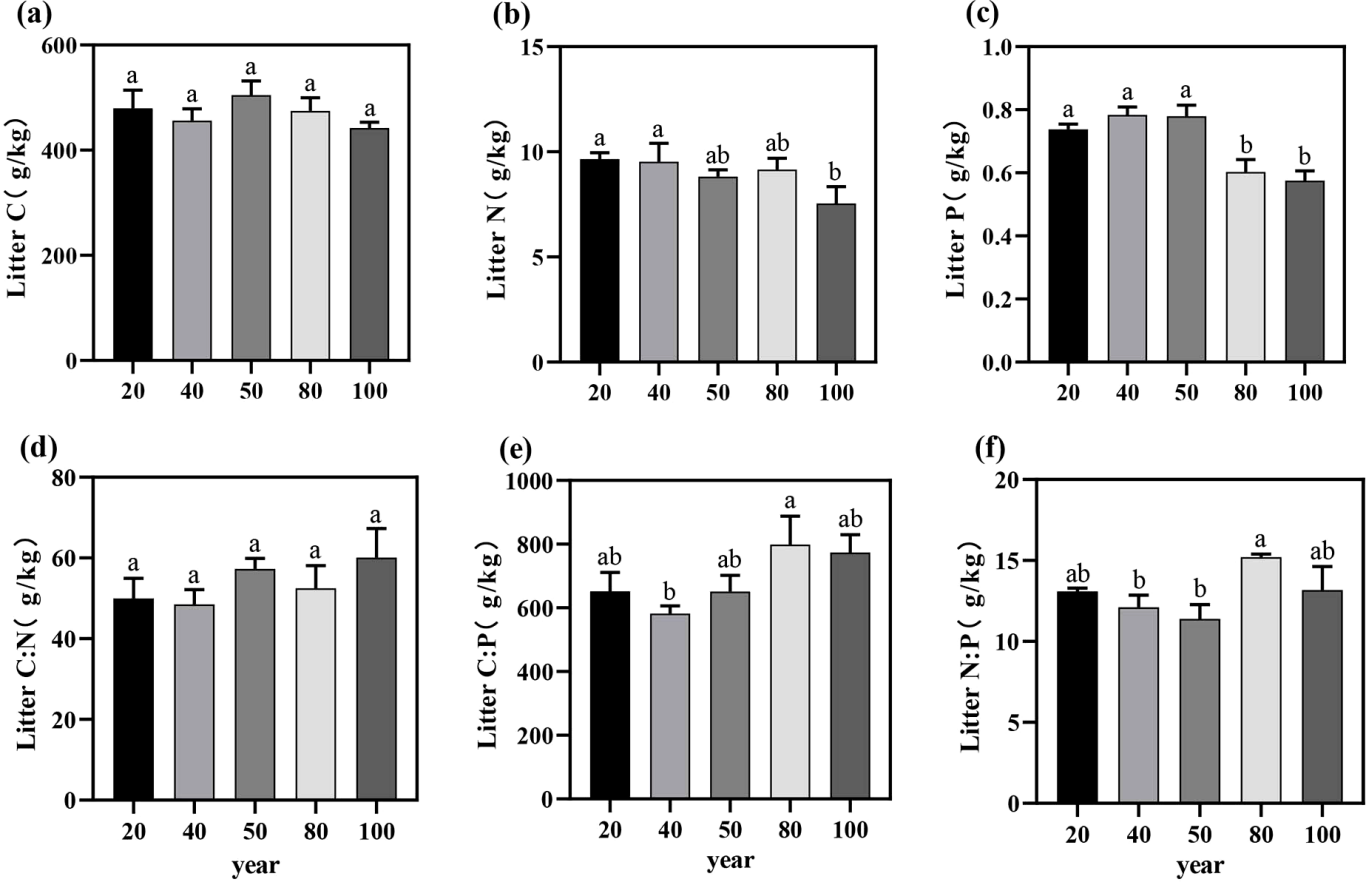
The variation trends of C, N, and P nutrient contents **(a–c)** and the C:N:P stoichiometric ratios **(d–f)** in the green leaves of *T. grandis* across different stand ages. Different letters indicate significant differences in leaf nutrient content among different stand ages (P < 0.05). Data without significant differences were not specifically labeled.

The leaf C, N, and P contents in the study area ranged from 334.42 ± 60.6 to 427.68 ± 36.15 g/kg, 11.75 ± 0.08 to 14.37 ± 1.07 g/kg, and 1.06 ± 0.22 to 1.46 ± 0.3 g/kg, respectively (**Figures 1a–c**). The contents of C, N, and P in leaves exhibited an initial increase followed by a decrease with stand age; however, no significant differences were observed among different stand ages (P > 0.05; **Table 1**). Similarly, the C:N:P ecological stoichiometric ratios did not show significant differences across stand ages (P > 0.05; **Table 1**), but similar variation trends were observed across the entire age range.

**Table 1:** Significance tests were conducted on the C, N, and P contents and the C:N:P ecological stoichiometry of *Torreya grandis* leaves, litter, and soil.

Index	Leaf			Litter		Soil-10cm		Soil-20cm	
	DF	F	P	F	P	F	P	F	P
C	4	1.65	0.236	0.906	0.496	1.294	0.336	2.994	**0.073**
N	4	4.91	**0.019**	1.884	0.19	2.300	0.130	3.532	**0.048**
P	4	2.313	0.129	10.769	**0.001**	2.001	0.170	4.515	**0.024**
C:N	4	1.641	0.239	0.939	0.48	0.964	0.468	0.976	0.463
C:P	4	2.383	0.121	2.353	**0.000**	6.702	**0.007**	4.353	**0.027**
N:P	4	2.219	0.14	3.261	0.059	6.723	**0.007**	5.484	**0.013**

P-values < 0.05 were marked in bold.

### 3.2 Nutrients contents and stoichiometric characteristics of litter

**Figure 2:**
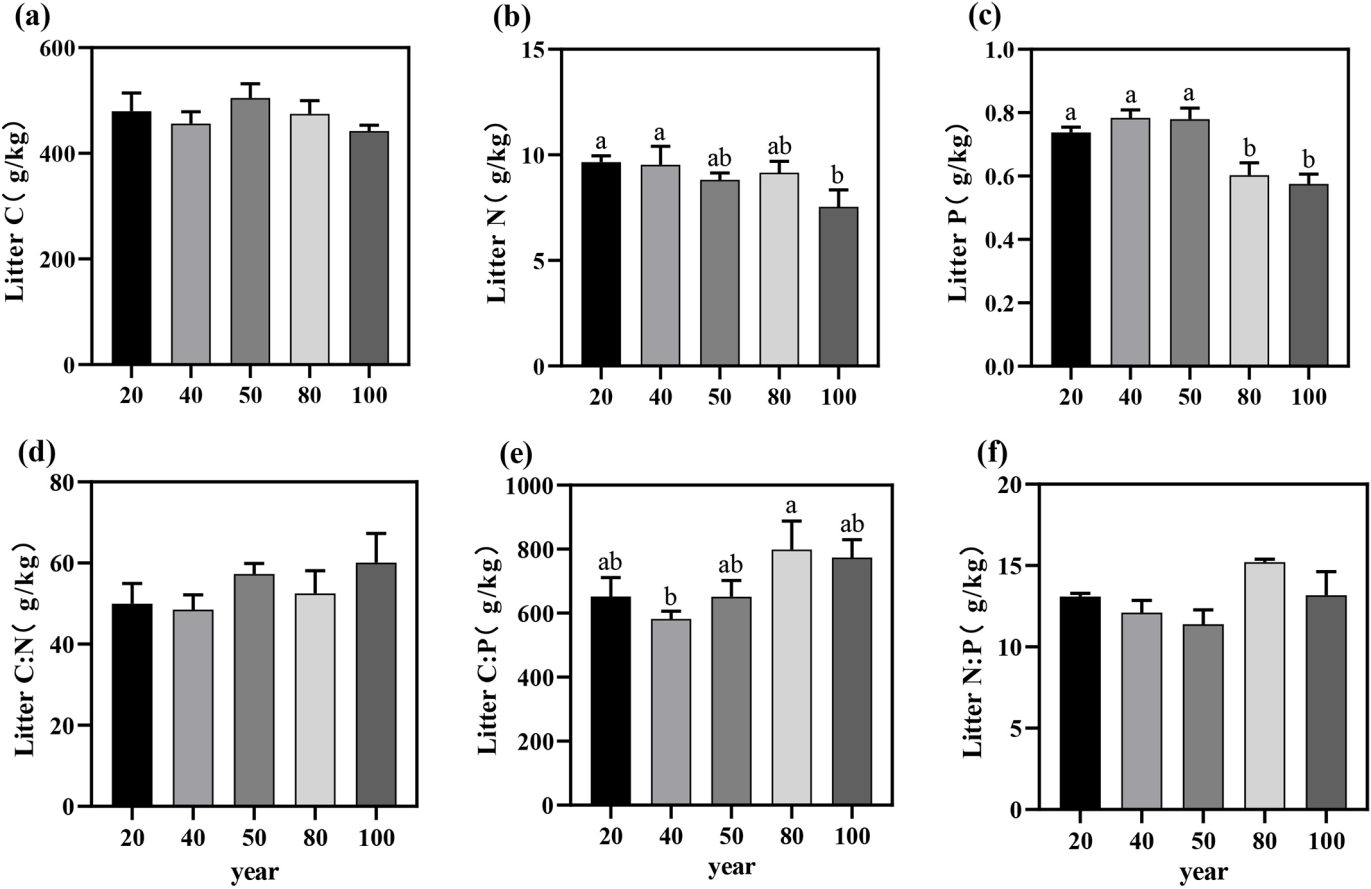
The C, N, and P nutrient contents **(a–c)** and the litter C:N:P stoichiometric ratios **(d–f)** of *Torreya grandis* leaf litter and their variation trends across different stand ages. Different letters indicate significant differences in leaf litter nutrient content among different stand ages (P < 0.05). Data without significant differences were not specifically labeled.

The C, N, and P contents in the litter within the study area ranged from 442.15 ± 19.16 to 504.92 ± 46.59 g/kg, 7.55 ± 1.39 to 9.66 ± 0.51 g/kg, and 0.58 ± 0.05 to 0.78 ± 0.04 g/kg, respectively (**Figures 2a–c**). With increasing stand age, the C and N contents in the litter exhibited an overall declining trend, though no significant differences were observed among different stand ages (P > 0.05; **Table 1**). However, the P content in the litter was significantly higher in the near-mature forest than in the mature forest (P < 0.05).

The C:N, C:P, and N:P ratios in the litter ranged from 48.5 to 57.31, 651.09 to 799.09, and 11.39 to 15.20, respectively (**Figures 2d–f**), with no significant differences in the C:N:P ecological stoichiometric ratios across stand ages (P > 0.05; **Table 1**). Overall, the nutrient contents and stoichiometric ratios in the litter exhibited similar trends with stand age, demonstrating a highly consistent pattern.

### 3.3 Nutrients contents and stoichiometric characteristics of soil

**Figure 3:**
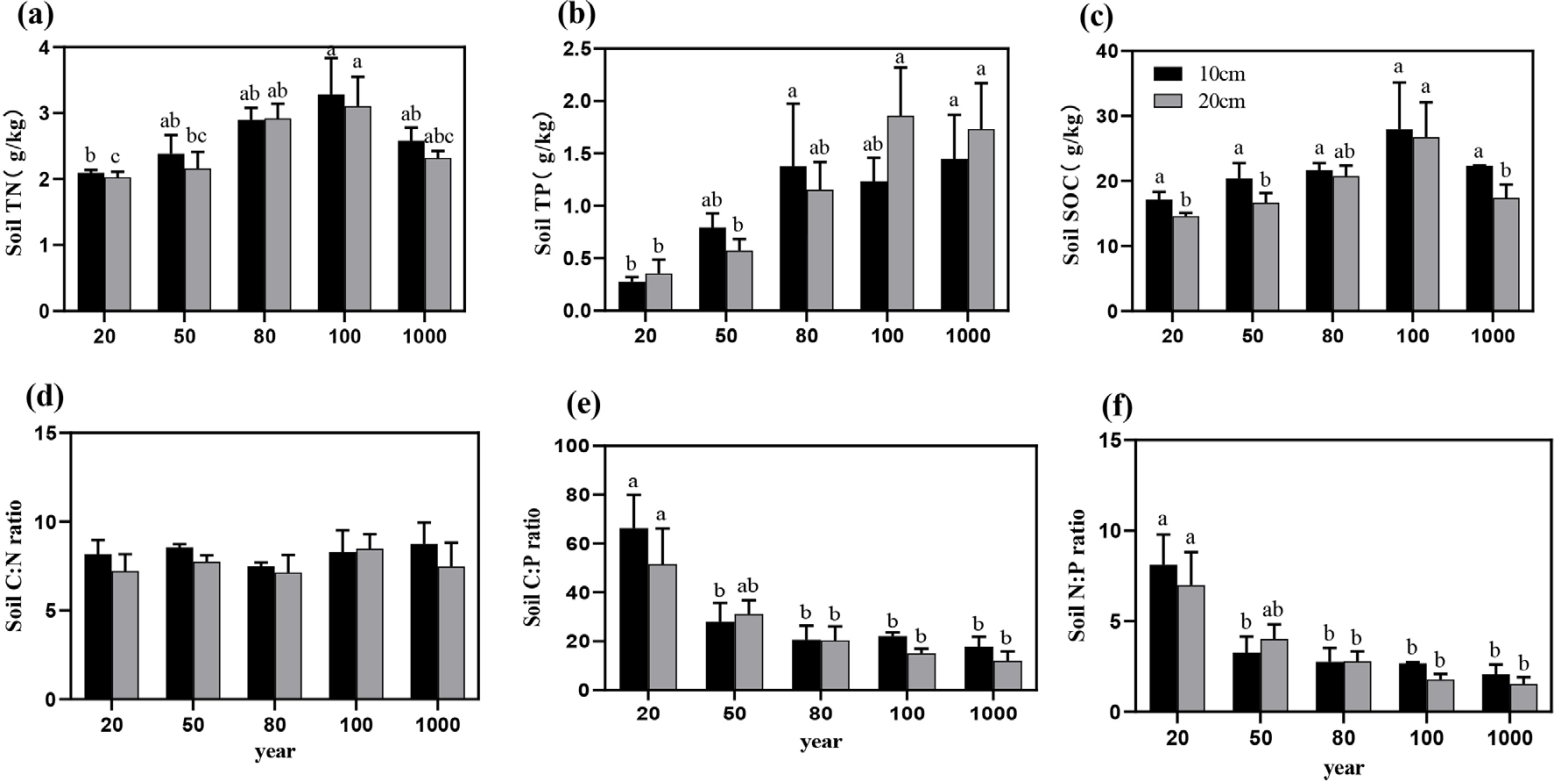
The variation trends of soil C, N, and P nutrient contents **(a–c)** and soil C:N:P stoichiometric ratios **(d–f)** across different forest ages in the 10-20 cm soil layer. Different letters indicate significant differences in soil nutrient content among different stand ages within the same soil layer (P < 0.05). Data without significant differences were not specifically labeled.

The contents of soil C, N, and P generally increased with stand age and exhibited significant differences (P < 0.05; **Table 1**). In the 10 cm soil layer, the C, N, and P contents ranged from 17.12 ± 2.05 to 27.95 ± 12.44 g/kg, 2.09 ± 0.08 to 3.28 ± 0.96 g/kg, and 0.27 ± 0.08 to 1.45 ± 0.73 g/kg, respectively (**Figures 3a–c**). In older *T. grandis* stands (mature, over-mature, and thousand), the soil C, N, and P contents were significantly higher than those in the 20-year-old stands (P < 0.05).

In the 20 cm soil layer, the C, N, and P contents ranged from 14.59 ± 0.83 to 26.75 ± 9.24 g/kg, 2.03 ± 0.14 to 3.11 ± 0.77 g/kg, and 0.36 ± 0.22 to 1.86 ± 0.79 g/kg, respectively. Similarly, in stands with greater stand ages (mature, over-mature, and thousand), nutrient contents were higher and significantly higher than those in the young stands. (P < 0.05) (**Figure 3d**).

The soil C:P and N:P ratios exhibited a consistent increasing trend with stand age (**Figures 3e, f**), with significant differences observed among different stand ages (P < 0.05; **Table 1**). However, the soil C:N ratio did not show significant differences across stand ages (P > 0.05, **Figure 3d**).

### 3.4 Reabsorption of nutrients in leaves

**Figure 4:**
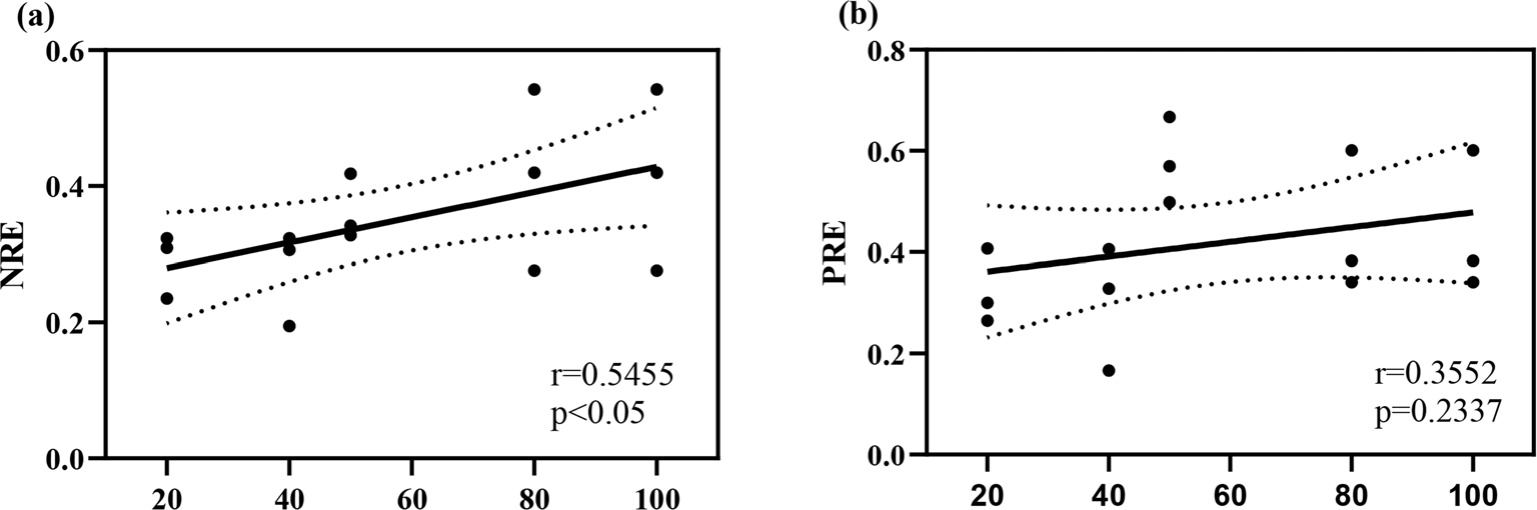
The linear regression curve of nutrient resorption in *T. grandis* leaves. Panel **(a)** represents the total nitrogen nutrient reabsorption rate, and panel **(b)** represents the total phosphorus nutrient reabsorption rate.

The results of the study (**Figure 4**) indicate that NRE varies significantly across different stand ages (P < 0.05), while PRE does not show significant differences (P > 0.05). As stand age increases, the resorption mechanisms for N and P in *T. grandis* plantations differ. In the young stand stage of *T. grandis* plantations, N resorption efficiency is relatively low but gradually increases with stand age.

### 3.5 Relationship among nutrient contents of leaf, litter, and soil

**Figure 5:**
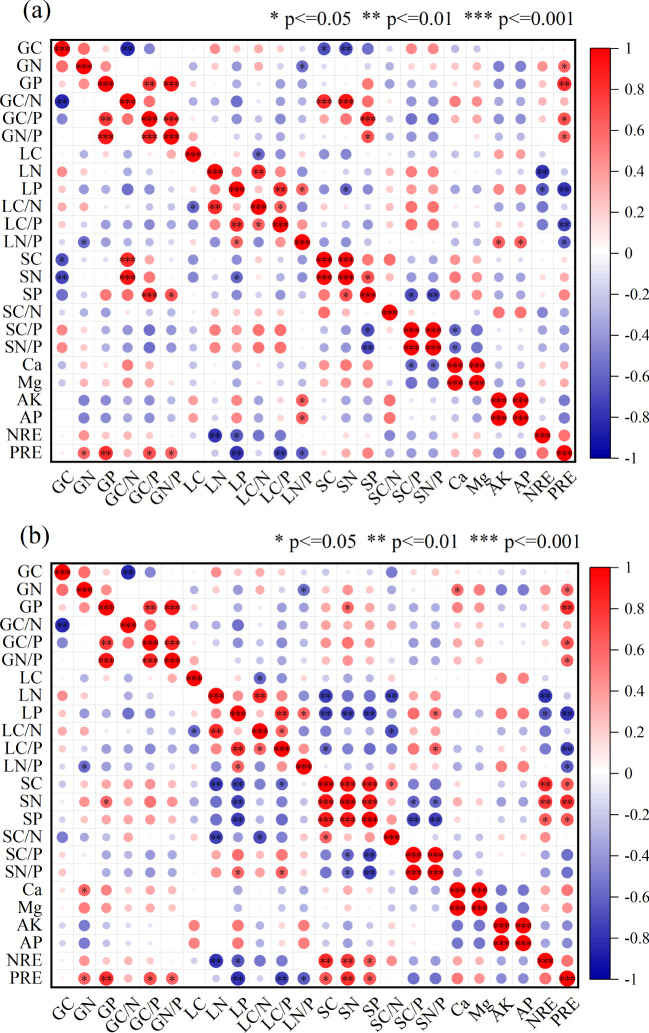
Pearson correlation heatmap of C, N, P contents and ratios, soil physicochemical properties, and nutrient resorption in the “leaf-litter-soil” continuum of *T. grandis* plantations. Here, G, L, and S represent green leaves, litter leaves, and soil, respectively. * indicates significant correlation at the 0.05 level (P < 0.05, two-tailed); ** indicates significant correlation at the 0.01 level (P < 0.01, two-tailed); *** indicates significant correlation at the 0.001 level (P < 0.001). Panel **(a)** represents soil at 10 cm depth, and panel **(b)** represents soil at 20 cm depth.

The nutrients C, N, and P exhibited the most significant correlations within the 20 cm soil layer (*P* < 0.01) (**Table 2**). Leaf C was positively correlated with N (P < 0.05). Litter P was correlated with soil C and N in the 10 cm layer (P < 0.05) and with C, N, and P in the 20 cm layer (P < 0.01). In the nutrient content of litter and soil stoichiometric ratios, Litter P was significantly correlated with the soil N:P ratio in the 20 cm soil layer (*P* < 0.01) (**Figure 5**). Soil N in the 10 cm layer was positively correlated with leaf C:N and C:P. In the soil-litter stoichiometric ratios, soil C at 20 cm negatively correlated with litter C:P (P < 0.05). Litter P showed a significant negative correlation with soil nutrients (P < 0.01). Soil C was positively correlated with C:N, while soil N and P were negatively correlated with C:P and N:P (P < 0.01). PRE was positively correlated with leaf and soil nutrients, but negatively correlated with litter nutrients. NRE was positively correlated with stand age, while PRE had a weaker correlation.

**Table 2:** The correlation between C, N, and P nutrient contents in green leaves, litter, and soil.

Item		Leaf			Litter			Soil-10cm			Soil-20cm		
		C	N	P	C	N	P	C	N	P	C	N	P
Leaf	C	1											
	N	0.575*	1										
	P	0.329	0.386	1									
Litter	C	0.418	0.1	-0.254	1								
	N	0.039	-0.354	0.057	0.007	1							
	P	-0.054	-0.279	-0.364	0.454	-0.011	1						
Soil-10cm	C	-0.404	-0.079	0.268	-.546*	-0.229	-.611*	1					
	N	-0.414	0.121	0.154	-0.486	-0.393	-.607*	0.911**	1				
	P	-0.511	-0.143	-0.075	-0.418	-0.171	-0.368	0.536*	0.671**	1			
Soil-20cm	C	-0.196	0.032	0.193	-.557*	0.004	-.739**	0.707**	0.764**	0.661**	1		
	N	0.096	0.368	0.432	-0.354	-0.318	-.696**	0.750**	0.775**	0.479	0.764**	1	
	P	-0.154	-0.032	0.35	-0.346	-0.114	-.696**	0.536*	0.479	0.643**	0.568*	0.571*	1

*Correlation is significant at the 0.05 level; **Correlation is significant at the 0.01 level.

**Figure 6:**
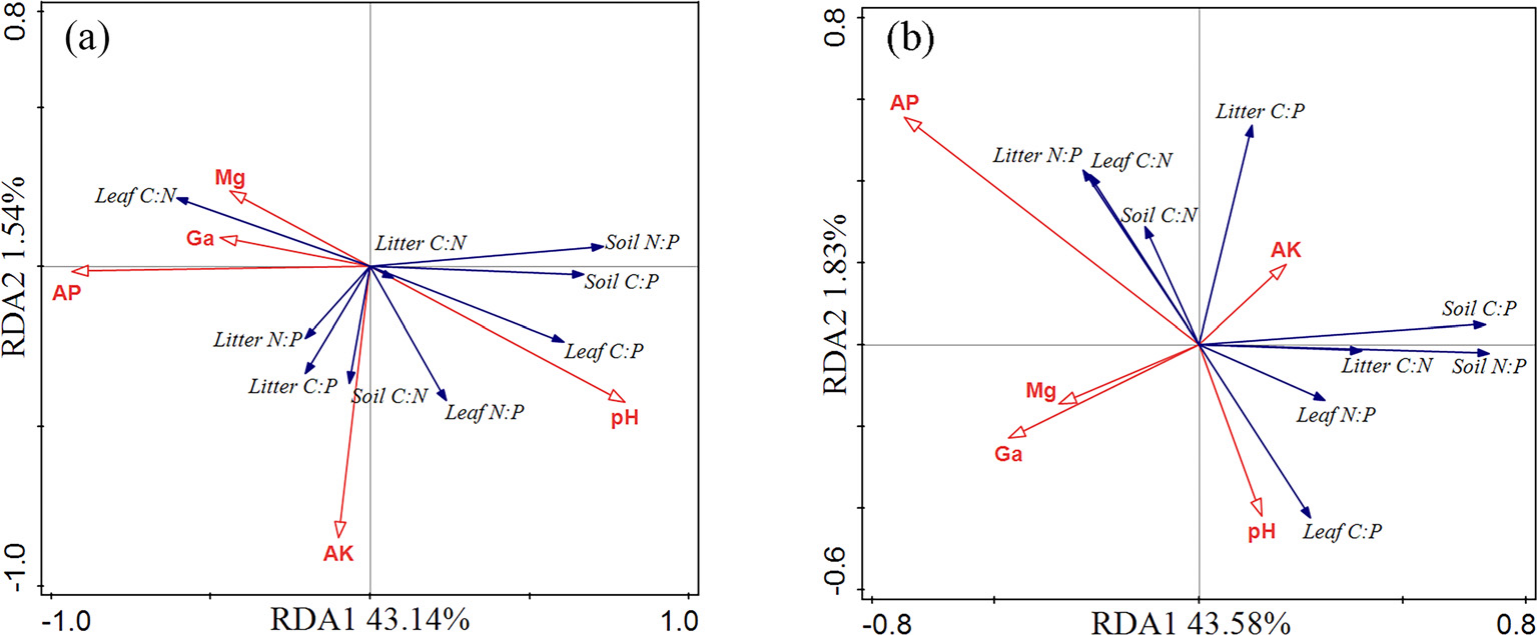
The relationship between ecological stoichiometry of leaf-soil-litter C:N:P and soil physicochemical properties. AK, available potassium; AP, available phosphorus; Ga, exchangeable calcium; Mg, exchangeable magnesium. Panel **(a)** represents redundancy analysis in the 10 cm soil layer, and panel **(b)** represents redundancy analysis in the 20 cm soil layer.

As indicated by the RDA analysis results, soil physicochemical properties (AP, AK, pH, Ca, and Mg) accounted for 50.9% of the total variation in the data (**Figure 6a**), with axes 1 and 2 accounting for 43.14% and 1.54%, respectively. AP had a much greater influence on leaf, litter, and soil C:N:P ratios than pH, AK, Ca, and Mg. However, axis 1 and axis 2 explained 43.58% and 1.83% of the total variation, respectively (**Figure 6b**), with AP and AK again having a significantly greater impact on the C:N:P ratios than pH, Ca, and Mg.

### 3.6 Seeds nutrient composition at different forest ages

With increasing stand age, the nutrient composition in *T. grandis* seeds initially declines and then stabilizes (**Table 3**), with significant differences observed across different stand ages (P < 0.05). Among the primary nutrients, the concentration of crude fat exhibits significant variation with stand age, peaking in the mature forest. Furthermore, all nutrient components reach higher levels in the near-mature or mature forests, except for ginsenoside acid.

**Table 3:** Nutrient composition and crude fat content of seeds at different stand ages.

Tree age	Nutrient components		Fatty acid composition in crude fat				
	Crude fat	Crude protein	OA	LA	LIA	S11EA	AUA
Young	45.8 ± 2.09a	11.5 ± 0.1b	36.65 ± 2.01b	39.14 ± 1.69a	0.46 ± 0.02a	0.63 ± 0.03c	8.34 ± 0.3a
Near-mature	45.53 ± 3.01a	12.7 ± 1.04ab	42.14 ± 1.27a	34.46 ± 1.34b	0.44 ± 0.01ab	0.76 ± 0.06ab	7.84 ± 0.09a
Mature	39.97 ± 4.1a	13.73 ± 0.59a	44.11 ± 1.61a	31.93 ± 1.49b	0.42 ± 0.03b	0.86 ± 0.03ab	8.19 ± 0.64a
Over-mature	43.43 ± 2.85a	11.93 ± 1.3b	41.33 ± 1.53a	34.72 ± 1.74b	0.42 ± 0.02b	0.73 ± 0.07bc	7.9 ± 0.19a
Thousand	40.17 ± 4.45a	11.53 ± 0.61b	41.66 ± 1.87a	34.68 ± 1.36b	0.43 ± 0.01ab	0.67 ± 0.02c	7.71 ± 0.45a

OA, oleic acid; LA, linoleic acid; α-LIA, α-linolenic acid; S11EA, cis-11-Eicosenoic acid; AUA, auropic acid. Different lowercase letters indicate significant differences at P < 0.05 level.

## 4 Discussion

### 4.1 Nutrient characteristics and stoichiometric differences

C、N and P are key limiting nutrients for plant growth. In most terrestrial ecosystems, these three elements play important roles in regulating plant growth, reproduction, and metabolism (Reich et al., 1997; Han et al., 2005). Leaf C (374.69 ± 55.21 g/kg) content in our research is significantly lower than averaged values reported in coniferous forests (484.5 g/kg) (Tang et al., 2018), global plant communities (461.60 g/kg) (Elser et al., 2000) and Chinese forests (455.00 g/kg) (Tang et al., 2018). At the same time, the leaf N (13.07 ± 1.19 g/kg) and P (1.19 ± 0.22 g/kg) concentrations are lower than the global and Chinese plant community averages (18.6 g/kg and 1.21 g/kg) (Han et al., 2005). The soil C (20.56 ± 6.10 g/kg) contents in the *T. grandis* plantation is lower than the global (25.71 g/kg) and national (29.51 g/kg) averages (Tian et al., 2010; Tao et al., 2016). The soil in the study area is sandy loam, which has a low C retention capacity, potentially affecting the soil C accumulation (Lal, 2004). The soil P (1.09 ± 0.73 g/kg) contents in the *T. grandis* plantation is higher than the national (0.56 g/kg) averages (Sun et al., 2019). Although a certain amount of P is present in the study area, it is typically bound to iron, aluminum, or calcium compounds in the soil, making it difficult for plants to absorb, limiting for plant uptake (Hinsinger, 2001), resulting in low P availability to *T. grandis*. High N deposition in the subtropical region and fertilization by farmers contribute to N accumulation in the soil (Zhu et al., 2015), leading to a slightly higher soil N content (2.58 ± 0.60 g/kg) than global (2.1g/kg) and national (2.3 g/kg) averages (Tian et al., 2010). In addition, soil C:P is used to reflect the effective availability of P (McGroddy et al., 2004), while soil N:P is commonly used to assess N and P nutrient limitations (Imaya et al., 2010). In this study, the average soil C:P and N:P ratios were 28.47 and 3.60, respectively, both lower than the national average levels (C:P ≈ 61.0, N:P ≈ 5.1) (Liu et al., 2010). The low C:P ratio may indicate P limitation in the ecosystem, which is likely due to N fixation causing P deficiency (Bell et al., 2014). Thus, high N and low P are the primary factors contributing to the significant changes in the soil C:P and N:P ratios in this region. The nutrient uptake by plants in the Chinese fir plantation is limited due to soil nutrient constraints. Under nutrient limitations, plants tend to adjust the nutrient content in their leaves to reduce competition with other species. For instance, some studies have indicated that in low-P soils, plants optimize resource use by adjusting nutrient allocation between roots and leaves, thereby reducing the N and P content in leaves (Li et al., 2016). As an economically valuable species, *T. grandis* demonstrates distinct ecological adaptability and nutrient utilization strategies compared to other conifers like pines or firs. These differences likely result in variations in leaf C, N, and P concentrations, reflecting unique adaptive mechanisms developed through long-term evolutionary processes (Wang et al., 2011b).

Research indicates that in the *T. grandis* plantation continuum (leaf-litter-soil), N and P concentrations are higher in the leaves than in the litter and soil. As the primary sites of assimilation and metabolism, leaves accumulate nutrients by fixing C and synthesizing organic compounds through photosynthesis (Zhang et al., 2018). Therefore, the N content in leaves exceeds that in the litter and soil. Before senescent leaves fall, plants resorb some N and P back into living organs (such as branches or roots) to minimize nutrient loss, leading to significantly lower N and P concentrations in the litter compared to the leaves (Killingbeck, 1996). Litter decomposition is a key nutrient source for the soil, but its nutrient input is somewhat reduced. As the primary metabolic organ, leaves retain higher levels of N and P. Through nutrient resorption, *T. grandis* lowers the N and P concentrations in the litter and soil, resulting in relatively lower nutrient content in the soil.

The growth rate hypothesis suggests that the C:N and C:P ratios are often inversely related to plant growth rate, reflecting vegetation productivity to some extent (Wang et al., 2011b). In this study, the average C:N and C:P ratios for *T. grandis* leaves were 28.7 ± 3.55 and 322.49 ± 57.43, respectively. These values are lower than those (C:N (40.4) and C:P (728.0)) for subtropical artificial evergreen coniferous forests in China (Wang et al., 2011b), indicating that *T. grandis* plantations, when faced with nutrient limitations, demonstrate adaptability to nutrient stress by optimizing nutrient absorption and allocation strategies, leading to more efficient use of N and P (Zhang et al., 2017). Previous studies have shown that the leaf N:P ratio in plant biomass reflects the relative N or P limitation at the community level (Güsewell, 2004). Previous findings suggest that an N:P ratio > 16 indicates P limitation, while an N:P ratio < 14 indicates N limitation. At intermediate values, plant growth is constrained by both N and P, depending on specific plant conditions (Reich, 2005; Fan et al., 2015). Relying solely on the N:P ratio to assess nutrient limitations in plant biomass production is insufficient; additional criteria are necessary. For instance, N limitation is indicated when N concentrations in green leaves fall below 20 g/kg, while P limitation occurs when P concentrations are below 1 g/kg (Zeng et al., 2017). In this study, the leaf N:P ratio was 11.25, with leaf N concentrations below 20 g/kg and P concentrations below 1 g/kg at 100 years (**Figure 1**). These results indicate that *T. grandis* plantations are primarily N-limited. During the fast-growing phase, N availability in the plantation is lower than in young and old forests, suggesting N as a limiting factor for growth. As the plantation matures, N limitation may shift to a combined N and P limitation. Under P-limited conditions, plants enhance P re-use by improving root absorption efficiency and increasing leaf P resorption, resulting in lower P content in senescent leaves (Liu et al., 2020). Moreover, the N:P ratios in both green and senescent leaves showed significant correlations with the P concentrations in both leaf types (**Figure 5**), consistent with previous research indicating that P limitation increases during the development of the plantation (Yan et al., 2018; Deng et al., 2019). The C:N and C:P ratios in litter can also reflect the supply of N and P in the soil (Tong et al., 2021).Studies have shown that nutrient release from litter reaches a critical threshold when the C:N ratio falls below 40 and the C:P ratio falls below 600, indicating effective release of N or P from litter (Parton et al., 2007). In this study, the average C:N ratio in the litter was 54.97 ± 8.92, above the C:N release threshold, while the C:P ratio averaged 719.13 ± 96.5, exceeding the C:P release threshold. These results suggest that the N and P content in *T. grandis* litter is not effectively released. It is inferred that the joint limitation of N and P in the growth of *T. grandis* may be due to the ineffective release of N from litter and the deficiency of P in the soil.

### 4.2 The influence of stand age on nutrients and stoichiometry

This study found that there were no significant differences in leaf C content across different stand ages, possibly due to the influence of environmental factors in the study area, and that leaf C content exhibits relatively stable characteristics. However, the contents of N and P were significantly affected, indicating that *T. grandis* plantations exhibit different ecological adaptation strategies at various developmental stages (Zhang et al., 2019). Although stand age has no significant effect on leaf N and P contents, their levels fluctuate with increasing stand age and reach the highest values in mature forests. In contrast, the C, N, and P contents in litterfall decrease with stand development, reflecting a conservative nutrient utilization strategy adopted by *T. grandis* during stand succession (He et al., 2024). The highest nutrient content in mature forests suggests that *T. grandis* plantations have increased organic matter in leaves and greater C storage capacity during the transition from young to mature forests. Another possible explanation is changes in photosynthetic characteristics, as plant age can influence photosynthetic performance (Bielczynski et al., 2017). The nutritional composition of *T. grandis* seeds (**Table 3**) also shows that fat and protein contents are higher in the mature forests range compared to other stand ages. It gradually stabilizes with increasing stand age, consistent with our hypothesis, the nutrient acquisition and transformation capacity of Chinese fir did not decline with increasing stand age. Soil C, N, and P contents increase with forest age, primarily due to litter decomposition and nutrient release. Additionally, as tree growth progresses, the rise in root exudates fosters soil organic matter formation, enhancing the storage of C, N, and P in the soil (Shi et al., 2021). Although the nutrient content of litter did not differ significantly with increasing stand age, the N and P contents in the litter exhibited an overall decreasing trend, while the C:N and C:P ratios showed an increasing trend as stand age increased, indicating a slower decomposition rate of litter in older forests, leading to a slower rate of nutrient replenishment to the soil (Yuan et al., 2024). Although nutrient concentrations in the litter decline with increasing stand age, the total amount of litter increases, which, through the cumulative effect, replenishes the soil nutrients (Li et al., 2023). As stand age increases, *T. grandis* maintains leaf nutrient stability through resorption mechanisms, while nutrient release and accumulation in the litter supply nutrients to the soil. The coupling of leaves, litter, and soil sustains the dynamic balance of the *T. grandis* plantation ecosystem.

This study found that the variation in leaf C:N:P ratios at different stand ages may be related to changes in nutrient demand during the growth of *T. grandis*. Although leaf C:N:P ratios do not differ significantly among stand ages, the rapid growth rate in the mature stand stage results in the lowest leaf C:P and N:P ratios (Pan et al., 2020). After reaching the mature forest stage, as growth rate slowed down, C:P and N:P ratios slowly increased, which aligns with the growth rate hypothesis theory, as confirmed by previous studies (Chen et al., 2018; Tong et al., 2019; Wu et al., 2020). Therefore, during the young and mature forest stages, *T. grandis* gradually reduced the C:N, C:P, and N:P ratios in the leaves through enhanced nutrient recovery efficiency and optimized nutrient allocation (**Figures 1d, f**). In contrast, during the over-mature forest stage, as nutrient competition intensified, soil nutrient supply became inadequate, and adjustments in nutrient uptake and utilization strategies occurred, leading to an increase in these ratios. Litter C:N and C:P ratios are typically used to reflect the efficiency of N and P utilization and plant growth rate. Studies have shown that the C:N:P ratios of litterfall do not differ significantly among stand ages. However, with increasing stand age, the C:N and C:P ratios of litterfall in *T. grandis* plantations increase, while the N:P ratio decreases but exhibits an increasing trend in the mature stand stage. This suggests that as stand age increases, growth enters a more stable phase, and nutrient demands for N and P decrease, leading to a relative reduction in their concentrations in the litter. However, the C content in the leaf was higher during the mature stage, causing the C content in the litter to increase, which in turn led to an upward trend in the litter C:N and C:P ratios with increasing stand age (Gartner and Cardon, 2004). Furthermore, as stand age increased, soil C:P and N:P ratios showed a downward trend. This was mainly due to the slow accumulation of P in the litter and the gradual insufficiency of soil P supply with increasing stand age, which resulted in the limited availability of P in the soil, thus leading to a decrease in the soil C:P and N:P ratios (Cleveland and Liptzin, 2007). Based on the above results, the growth of *Torreya* is constrained by both N and P. In the early stages, N is the primary limiting factor, but as the stand develops, the limitation shifts to N and P co-limitation, with P becoming the dominant constraint in older stands. The results also indicated (**Figure 5**) that the soil C:P and N:P ratios were negatively or significantly negatively correlated with soil P content, suggesting that changes in soil P across different stand ages were the primary cause of the variations in soil C:P and N:P ratios (Liu et al., 2023).

### 4.3 Nutrient resorption characteristics at different stand ages

Compared to fresh leaves, the N and P content in senescent leaves of *T. grandis* plantations of various ages significantly decreased, reflecting the plant’s nutrient resorption characteristics (He et al., 2023). During the young stage (**Figure 4**), plants support rapid growth by enhancing PRE, whereas in the mature to overmature stages, as soil N availability decreases, plants rely more on improving NRE to sustain growth (Callesen, 2004). Since the N recycling process is influenced by both environmental conditions and the plants’ physiological characteristics traits (Mediavilla et al., 2014; Sun et al., 2016), NRE is likely regulated by the overall plant-soil system rather than solely by soil N concentration. The *T. grandis* plantation adjusts its nutrient conservation and utilization strategies as stand age changes. To maximize N and P use efficiency, older stands exhibit a more conservative P utilization strategy, achieved by increasing PRE and reducing leaf P concentration. In contrast, their N utilization strategy also becomes more conservative but through a different mechanism: enhancing NRE without lowering leaf N concentration. This phenomenon is consistent with previous studies on plant nutrient acquisition strategies along sand dune succession sequences (Hayes et al., 2014). Furthermore, total soil N shows a positive correlation with NRE, PRE, and the N:P ratio, and a negative correlation with N and P concentrations in senescent leaves (**Figure 5**). This indicates that the relationship between total soil N and leaf nutrient status (e.g., nutrient resorption and leaf nutrient concentrations) is closer, supporting the view proposed by previous studies that soil N has a positive effect on P resorption in the leaves of *T. grandis* plantations (Craig et al., 2015). The close interaction between plants and soil is critical for nutrient cycling and tree growth.

Across all stand ages, the PRE is always higher than the NRE (**Figure 4**), indicating that sustained P resorption within plants is more P than N resorption (Reed et al., 2012). The reasons for this disparity are as follows: Firstly, P has a higher reactivation ability compared to N. Studies have shown that P resorption occurs throughout the leaf’s entire lifespan, while N resorption mainly happens during leaf senescence prior to litterfall (Achat et al., 2018). Secondly, there is a lack of external P input in the *Torreya* forest, but a significant amount of external N input occurs through atmospheric N deposition. In subtropical regions, there is a large amount of N deposition, and experiments simulating N deposition in larch plantations have shown that external N input reduces leaf NRE (Sun et al., 2016). Thirdly, plants invest more photosynthates and energy to acquire soil-available P, while N bioavailability largely depends on soil microbial activity. Soil microbes mineralize organic N into inorganic N, which roots can absorb directly without energy expenditure. In contrast, only a small fraction of inorganic P is mineralized from organic forms, with most being rapidly adsorbed onto mineral surfaces or bound in inorganic precipitates (e.g., Fe, Al, or Ca) (DeLuca et al., 2015; Wu et al., 2020). Therefore, plants adopt a more conservative strategy for internal P cycling, which differs from the strategy used for N cycling (Reed et al., 2012). This divergence reflects the distinct strategies plants utilize for P and N resource acquisition.

Additionally, the nutrient allocation in *T. grandis* plantations is also reflected in the nutrient composition of its seeds. The oil, protein, starch, and other nutritional components, as well as the fatty acid composition of *T. grandis* seeds, are critical indicators influencing seed quality. The higher the protein and oil content, and the lower the starch content, the more delicate and crisp the seed’s texture becomes (Fang et al., 2021). According to the research on crude fat and crude protein, it was found that these components were relatively higher in near-mature and mature stands (**Table 3**). This trend aligns with the leaf C, N, and P nutrient contents, suggesting that during these two age stages, the accumulation of nutrients in the plants is greater, thus enabling sufficient nutrient allocation to the seeds. With increasing stand age, the nutrient composition of *T. grandis* gradually stabilizes without significant decline. This phenomenon can be attributed to the development of an efficient N and P uptake and nutrient allocation mechanism during its growth. Similar phenomena have been observed in other studies, where the leaves and root systems of *T. grandis* exhibited efficient N and P uptake and allocation capacities in trees older than 500 years (He et al., 2024). The stability of this mechanism plays a crucial role in maintaining the effective accumulation and redistribution of nutrients throughout the long-term growth of *T.* grandis. Therefore, the growth and quality of *T. grandis*, influenced by stand age, nutrient content, and their relative ratios, are important factors in determining the nutritional composition of the seeds.

## 5 Conclusions

This study reveals the significant influence of stand age on the C:N:P stoichiometry, nutrient dynamics, and ecological adaptability of *T. grandis* plantations. Nutrient concentrations in leaves, litter, and soil exhibit distinct patterns during stand development, reflecting shifts in nutrient limitation and allocation strategies. Young stands are primarily N-limited, transitioning to N-P co-limitation as the stand matures, with P limitation becoming more pronounced in overmature forests. *T. grandis* enhances PRE during the early stages and relies on higher NRE in overmature stands, demonstrating its conservative nutrient utilization strategy. Moreover, the nutrient composition of seeds is closely associated with leaf nutrient dynamics, indicating a tight coupling between nutrient allocation and seed quality. Overall, the growth and nutrient dynamics of *T. grandis* plantations are influenced by stand age and soil nutrient supply. Future management should prioritize soil P supplementation and improved nutrient cycling efficiency to promote sustainable development and enhance the ecological and economic value of plantations.

## Data Availability

The raw data supporting the conclusions of this article will be made available by the authors, without undue reservation.
